# Effects of early-life protein starvation on longevity and sexual performance of male medfly

**DOI:** 10.1371/journal.pone.0219518

**Published:** 2019-07-25

**Authors:** Stella A. Papanastasiou, James R. Carey, Nikos T. Papadopoulos

**Affiliations:** 1 Department of Agriculture, Crop Production and Rural Environment, University of Thessaly, Volos, Greece; 2 Department of Entomology, University of California, Davis, California, United States of America; Inha University, REPUBLIC OF KOREA

## Abstract

Using a well-established model species for demographic, behavioural and aging research, the Mediterranean fruit fly (*Ceratitis capitata*), we explored whether nutritional stress early in adult life affects the sexual performance and survival in older ages. To do so we established two different protein starvation (PS) protocols that included the elimination of proteinaceous diet either before or after sexual maturity of male medflies. The frequency of sexual signalling and the age of death were daily recorded. Sexual signalling is directly related with male mating success in this model system. PS early in adult life results in high mortality rates (similar to sugar-only fed males), which are gradually restored in more advanced ages. Provision of a proteinaceous diet following early-life PS increases straightaway male sexual signalling to levels similar with those having continuous access to proteinaceous diet. Switching diet regimes from a protein-free to a protein-rich one progressively compensates mortality rates. Apparently, males prioritize sexual signalling over lifespan. PS after attaining sexual maturity significantly reduces both longevity and sexual performance. Access to protein only early in life is insufficient to support lifetime energy-consuming behaviours such as sexual signalling. Continuous access to a proteinaceous diet determines both lifetime sexual performance and longevity. Early in life PS males prioritize the allocation of nutritional elements, when available, in sexual activities over soma-maintenance.

## Introduction

Nutrient intake by insects has been extensively studied regarding energy regulation in adult stages, its allocation to biological traits, and the effects on several life history parameters. Resource allocation to physiological functions (soma maintenance, growth, reproduction), during adverse and optimal nutritional conditions, is a genetically-controlled dynamic procedure that affects the fitness of various organisms [1, 2]. The competition between two or more physiological processes for the same pool of limited resources leads to physiological trade-offs. The ability to reallocate energetic resources between the two major features of life cycle (reproduction and survival) promotes fitness. Most of the studies supporting life-history trade-offs are rather deductive. The causal physiological mechanisms underpinning these complex procedures (i.e. nutrient ingestion, assimilation and allocation pathways to organismal functions, hormonal signalling) have only recently progressed [2]. Manipulation of the nutritional environment, commonly adopted either as dietary restriction (DR, decrease of total diurnal quantity and/or quality of diet intake), as caloric restriction (CR, decrease of energetic metabolites intake) or as protein starvation (PS, complete elimination of protein intake), is broadly applied on model-organisms towards resolving questions regarding nutrition, diseases, senescence and longevity [1, 3, 4]. Diet manipulations (DM) are usually achieved by taking into account the optimum daily availability of energy and basic nutrients. They are assessed through a wide variety of dietary protocols and combinations of nutritious levels that can be applied to different life stages or even by altering them in relation to age [5–9]. This extreme variability in diet protocols often leads to contradictory results that are difficult to interpret and draw safe conclusions.

Somatic responses to DM relate to evolutionary adaptations favouring organisms that can extend their lifespan during periods of scarce nutrient resources [10]. The well documented effects of DR to extend the lifespan of various organisms (fungi, nematodes, arthropods, insects and mammals) have been previously attributed to CR [11]. However, several recent findings using different model species support the importance of nutrients rather than calories for longevity extension in DM studies [12–18]. It is generally accepted that protein intake increases reproductive investment and decreases lifespan.

Furthermore, DR protocols may exert differential effects on the life span and reproductive investment of male and female insects [19, 20]. Previous studies report a sexual dimorphism in foraging choice of several insect species [20, 21]. Differential nutrient balance between the two sexes in relation to their developmental stages, mating status and reproductive effort has been revealed (reviewed in [21]). Allocation of nutrients under a DR protocol to reproduction and soma maintenance may vary between males and females resulting in distinct regulation of the cost of reproduction, and therefore longevity extension between the two sexes [20].

Early-life stress triggers physiological mechanisms and behavioural responses towards promoting survival and/or reproduction, which may be costly (increased morbidity and mortality) later in life [22]. In insect species where adults live several days or weeks, the energetic reserves accumulated during larval development are rarely sufficient to sustain reproductive performance. It is necessary for males of these species to find nutrient and energy resources in order to successfully engage in reproductive activities. However, foraging in nature can be difficult for adults, since long periods of no availability or scarce resources may prevail. This may result in low nutritional status of males by the time they reach sexual maturity. In fruit flies (Diptera: Tephritidae) and other holometabolous insects, although development is completed upon adult emergence, access to a high quality proteinaceous diet early in life promotes reproductive performance of both sexes including male sexual performance [23, 24]. However, the relationship between PS early in adult life and the performance of males at advanced ages, including possible associated costs (decreased longevity and/or sexual performance) has not been addressed.

The Mediterranean fruit fly has been intensively used as a model organism in demographic research. Female medflies exhibit two “modes” of ageing in relation to oviposition rates depending on their access to protein-rich diet. PS females stay in a waiting mode of low mortality and reproduction and upon access to proteinaceous diet turn into a reproductive mode in which mortality is very low at the onset of egg laying but accelerates as eggs are laid [25]. Furthermore, DR increases female survival when it is applied as a periodic availability of protein [5], as well as when a protein restricted diet is switched to a protein-rich one [25]. Male medflies that have access to a diet with moderate levels of protein, sugar and vitamins live longer than those with access to a diet with higher nutritional value or than males feeding a sugar-only diet [7]. Complete elimination of yeast hydrolysate from adult male diet results in detrimental effects on both longevity and reproduction (mating success, sexual signalling rates) [26–28]. Caloric restriction seems to have a neutral effect on lifespan of adult medflies with males outliving females [6, 29]. On the other hand, discontinuous starvation after various periods of access to a sugar-only or to a yeast-sugar diet causes higher increase in female than male lifespan [30].

Despite the available knowledge regarding the DM outcome on medfly life history traits, the effect of PS early in adult life on the survival and reproduction performance of male medflies has not been tested yet. Previous studies indicate that male sexual signalling constitutes a biomarker of aging and that protein availability in adult diet accelerates sexual maturation and increases the frequency of sexual signalling and average lifespan in male medflies [4, 28, 31, 32]. However, no information is available regarding the effect of PS early in life on the frequency of sexual signalling (major determinant of reproductive success) and on the lifespan of male medflies. We tested the hypotheses that (a) PS early in adult life of male medflies is associated with both reduced sexual signalling, and decreased longevity, and (b) nutritional “reserves” attained through early-life provision of a proteinaceous diet are invested towards sustaining and/or maximizing sexual performance rather than soma maintenance and mortality decrease in advanced ages.

## Materials and methods

### Flies and rearing conditions

Wild medflies that were obtained from infested fruits of untreated trees collected in a private orchard, in the area of Volos, Greece (39°19’12.40”N, 23°1’39.45”E) were used for establishing a small-scale rearing. No specific permission was required for the location and the activities taken place in our study either than the owner’s permission to enter his property and collect fallen fruit, as no endangered or protected species were involved. Sampling fruit is a common practice to obtain wild populations of medfly. In all trials we used F_1_ medflies reared and tested in standard laboratory conditions (25 ± 1°C, 65 ± 5% R.H., and a photoperiod of L14:D10 with photophase starting at 07:00 am). In detail, following emergence wild adults were kept in groups of approximately 200 individuals in wooden (30x30x30 cm), wire-screened cages, and had free access to water and a standard diet consisting of a mixture of yeast hydrolysate, sugar and water at a 1:4:5 ratio (YS). Females were allowed to oviposit on an artificial oviposition substrate (5 cm diameter hollow, plastic red-coloured hemisphere, pin-punctured with 40–50 evenly distributed holes, adjusted on the lid of a 5.5 cm diameter plastic Petri dish) [33]. Each cotton disk was kept in a 9 cm diameter glass Petri dish on a layer of sterilised sand where pupation occurred after the conclusion of larvae feeding. F_1_ pupae were sieved and groups of approximately 50 individuals were placed in 20x20x20 cm Plexiglas cages.

### Diet and dietary protocols

Within 24 hours after adult emergence 400 males were transferred in individual cages (400 ml plastic transparent caps) with free access to water and adult diet [either yeast hydrolysate, sugar and water at a 1:4:5 ratio (YS) or sugar and water at a 1:3 ratio (S)]. The YS diet consisted of yeast hydrolysate and pure sucrose (1:4 ratio by volume) whereas the S diet consisted of pure sucrose only. The yeast hydrolysate enzymatic (MP Biomedicals LLS, Santa Ana, CA, USA) contained 60% of water soluble protein along with carbohydrates, B complex vitamins and minerals. Any response to PS was mediated by the timing of absence/presence of yeast content in the dietary protocol.

**Fig 1:**
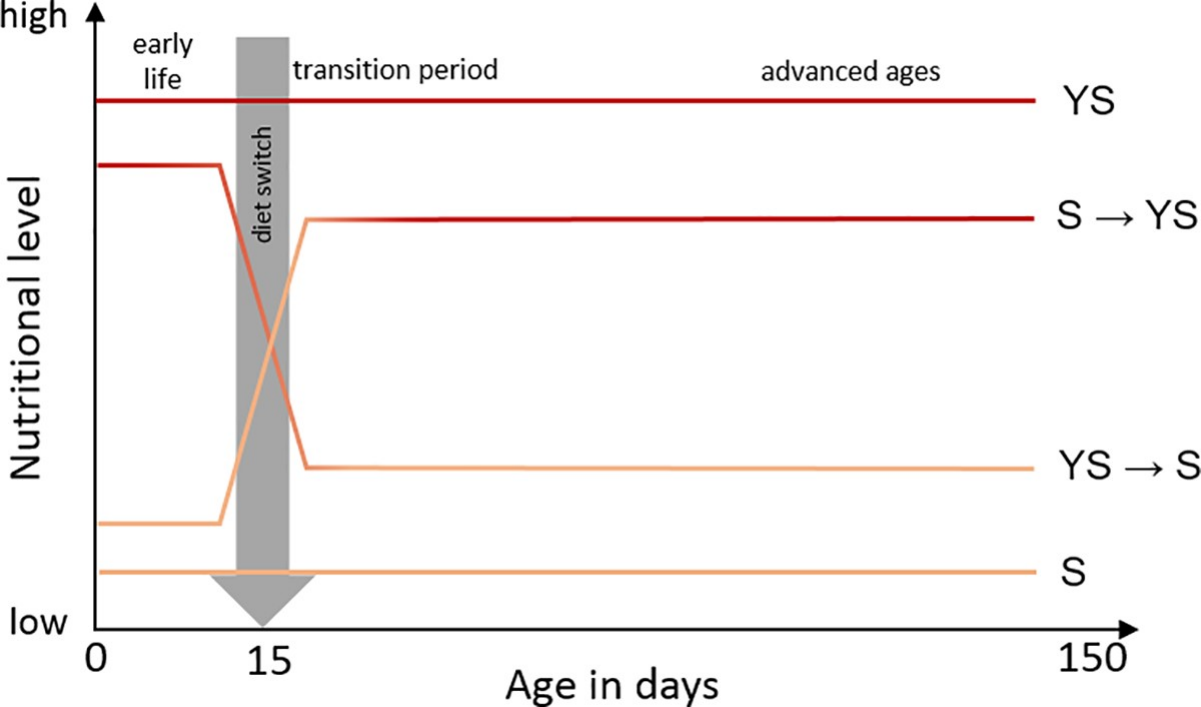
Graphic representation of the dietary protocol applied to male medflies. Y axis demonstrates the level of nutritional value in the diet (low: sucrose–S, high: yeast hydrolysate plus sugar–YS). X axis indicates the qualitative progression of male age expressed in days. Diet switch was applied at the age of 15 days that defines attainment of sexual maturity of male medflies.

Diet alteration from YS to S and from S to YS was applied to 50 randomly chosen males, from each cohort, at the age of 15 days that is known to define the conclusion of sexual maturity in wild male medflies (Fig 1). Consequently, 150 remaining males had constant access to YS diet and 150 more had constant access to S diet throughout life. Thereby, according to the described dietary regime, 4 groups of males were created: i) S throughout life [sugar-only (S) controls], ii) S until 15 days old then YS until death (S → YS), iii) YS throughout life [full-diet (YS) controls] and iv) YS until 15 days old then S until death (YS → S).

### Sexual signalling and mortality observations

Sexual signalling and mortality observations were performed at a total of 400 individually kept males assigned to 4 dietary treatments (see above). Individuals that died before the age of 15 days, when diet alteration was applied, were excluded from the analyses. Therefore, after the age of 15 days, we recorded on a daily basis the mortality and the sexual signalling of 141 YS males, 42 YS → S males, 147 S males and 47 S → YS males. Every 10 min between 10:00 am and 12:00 (total of 13 readings per day), when the diurnal sexual signalling peaks, we visually recorded whether or not the individually kept males exhibited sexual signalling (male is curling the abdomen upward while extruding the terminal end of the rectal epithelium)[34].

### Statistical analyses

Data analyses were performed using the statistical software JMP 9.0 (SAS Institute, Cary, NC, U.S.A.) and SPSS 24.0 (SPSS Inc., Chicago, IL, U.S.A.). The effect of dietary regime and of the frequency of sexual signalling on male survival was assessed through the non-parametric Cox proportional hazards model. We also used the Kaplan-Meier estimate to depict survival patterns and calculate percentiles survival estimates for each dietary treatment. Additionally, we used the Akaike Information Criterion (AIC) to assess a parametric model fit for our survival data. Testing the survival data of each of the four dietary treatments separately to define the model that fitted them most, we concluded that the Weibull distribution was the most appropriate to proceed with the analyses (ranking at the first two best fits). Therefore, the 2-parameter Weibull model was applied in order to estimate the effect of diet and its alteration on male survival [35].

We used the Repeated Measures ANOVA to assess the effect of age and diet regime on the mean daily sexual signalling, per 10-day interval, through the age of 100 days. The effect of protein starvation/provision early in adult life on the lifespan and the mean daily frequency of sexual signalling of male medflies was tested with Multivariate Analysis of Variance (MANOVA). Only males that lived longer than 15 days were included in the analysis and only the frequency rates of sexual signalling after the 15^th^ day of age were used for the calculation of average sexual signalling. Tukey’s HSD was used to compare the mean sexual signalling and the lifespan of males subjected to the 4 diet regimes.

## Results

### Effect of diet manipulation on male longevity

**Fig 2:**
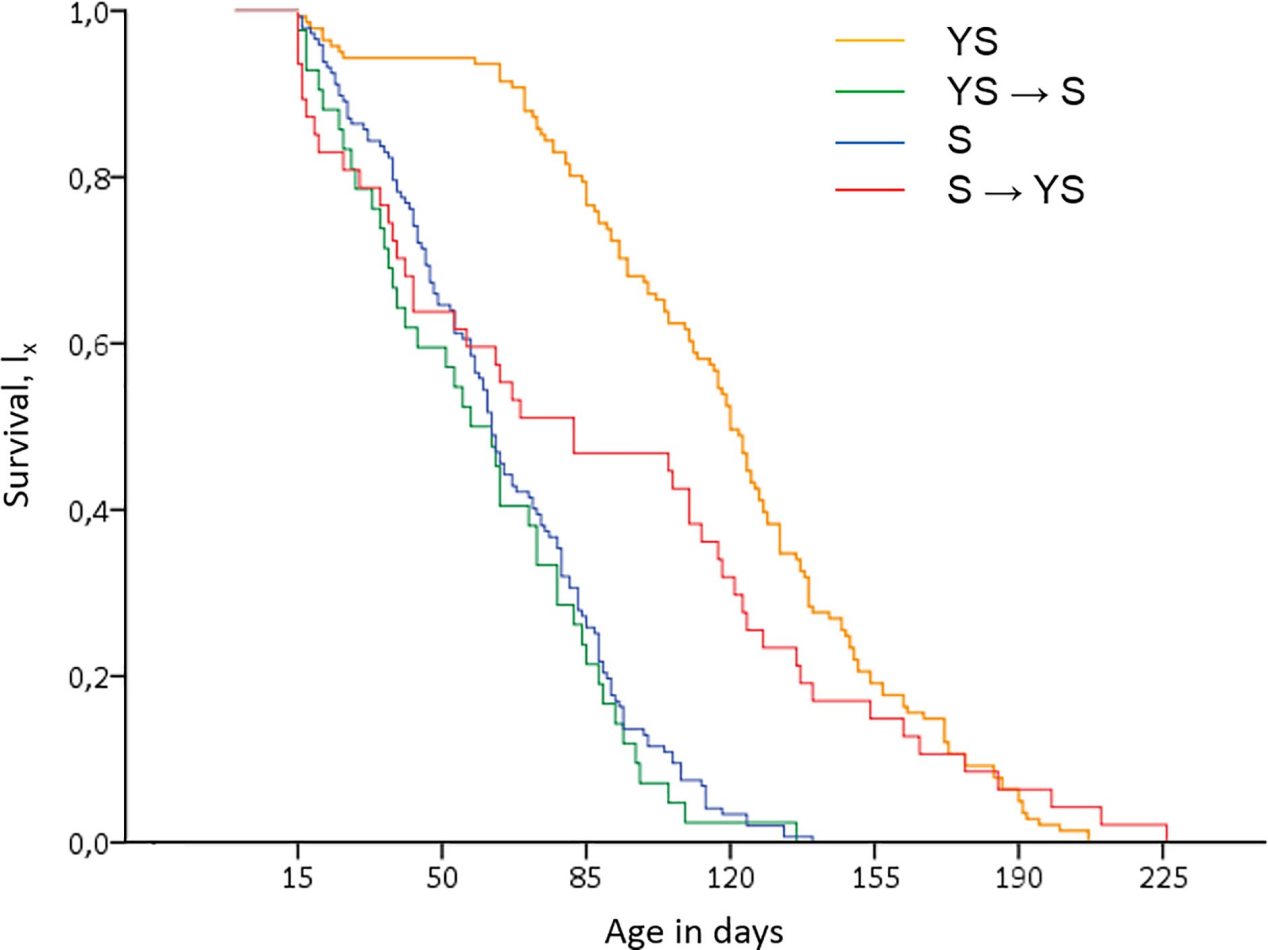
Kaplan-Meier survival estimates of males subjected to 4 different dietary regimes [141 males continuously fed yeast-sugar (YS), 42 males fed yeast-sugar until the age of 15 days then sugar (YS → S), 147 males continuously fed sugar (S), 47 males fed sugar until the age of 15 days then yeast-sugar (S → YS)] in relation to age.

The Cox proportional hazards model revealed that diet significantly affected male survival (Wald test *t* = 23.466, df = 3, *P* < 0.001) (Fig 2, Tables 1 and 2). This is also supported by MANOVA (*F* = 51.63, df = 3, 373, *P* < 0.001). Full diet control males (YS) outlived all other treatments. Sugar-only fed males (S) and YS → S males exhibited similar, notably shorter lifespans than the two other treatments (*P* < 0.05). Likewise, average lifetime sexual signalling and the interaction between the diet treatment and the sexual signalling were significant predictors of mortality rates (Wald test *t*_*signalling*_ = 8.576, df = 1, *P* = 0.003 and Wald test *t*_diet*signalling_ = 27.3639, df = 3, *P* < 0.0001) (Table 1).

**Table 1:** Variables of the Cox proportional hazards model for the sexual signalling and for the 4 dietary regimes of male medflies. Males continuously fed the yeast-sugar (YS) diet compose the baseline group.

Source of variance	*β*	St. error	Exp(β)	*P*
YS → S	0.864	0.356	2.372	0.015
S	0.859	0.271	2.361	0.002
S → YS	1.897	0.399	6.669	<0.001
Sexual signalling	-0.105	0.036	0.900	0.003
Sexual signalling * YS → S	0.163	0.098	1.177	0.095
Sexual signalling * S	0.101	0.066	1.106	0.130
Sexual signalling * S → YS	-0.286	0.071	0.751	<0.001

**Table 2:** Mean lifespan and percentiles of male medflies subjected to the four dietary protocols. Values followed by different letters are significantly different (Risk Ratio test following Proportional Hazards Fit Analysis, *P* < 0.001).

Diet treatment (Number of males)	Longevity parameters in days ± SE			
	Mean lifespan	Quartiles		
		25	50	75
YS (n = 141)	118.67 ± 3.64a	148 ± 4.56	120 ± 2.79	88 ± 4.26
S (n = 147)	65.03 ± 2.40c	88 ± 2.81	62 ± 2.14	43 ± 2.51
YS → S (n = 42)	59.57 ± 4.64c	84 ± 6.90	57 ± 6.02	35 ± 5.13
S → YS (n = 47)	88.66 ± 8.73b	128 ± 9.19	82 ± 28.05	37 ± 7.47

The parametric Weibull fitted hazard function produced similar results to that of Cox regression (S1 Fig, S1 Table). Fitted Weibull survival curves revealed that males subjected to the S → YS diet had higher mortality rates in younger ages compared to males continuously fed the YS diet. Significant shifts of deaths at earlier ages for the S → YS compared to YS diet regime are highlighted by the fitted Weibull density curves showing that the estimated probability of deaths peaked at approximately day 50 and 120, respectively (S1 Fig, S1 Table). Likewise, hazard rates of the S → YS diet regime are higher than the YS one approximately until day 110 when a switch over is recorded. Males seem to compromise longevity over reproduction after switching from a poor to a rich diet.

### Effect of diet manipulation on lifetime sexual signalling

**Fig 3:**
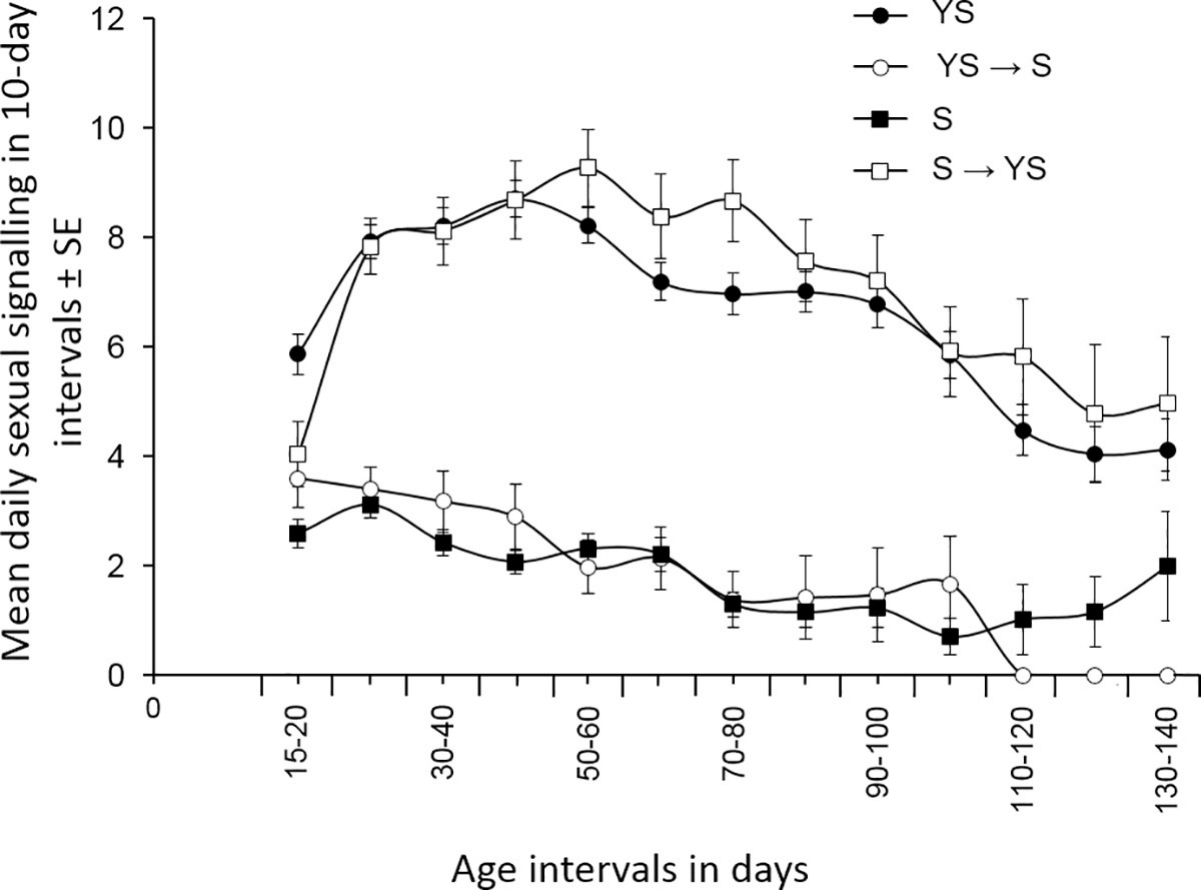
Mean daily sexual signalling per 10-day intervals (frequency of 13 daily observations per male in the respective time interval) until the age of 140 days of males subjected in 4 dietary regimes [continuously yeast-sugar (YS), yeast-sugar until the age of 15 days then sugar (YS → S), continuously sugar (S), sugar until the age of 15 days then yeast-sugar (S → YS)].

Diet quality and its alteration significantly affected male sexual signalling (MANOVA: *F* = 99.81, df = 3, 367, *P* < 0.001). Full diet control males (YS) exhibited the highest average daily rates of sexual signalling (5.56 ± 0.18) followed by S → YS males (5.01 ± 0.31), with no significant differences between them (Tukey’s HSD test *P* > 0.05). Sugar-only control males (S) exhibited the lowest average daily rates of sexual signalling (1.66 ± 0.17) that were similar to these of YS → S males (1.8 ± 0.33) (Tukey’s HSD test *P* > 0.05). The average daily sexual signalling of the two first groups of males (YS and S → YS) was significantly higher than that of the two latter groups (S and YS → S) (Tukey’s HSD test *P* < 0.05). The Repeated Measures ANOVA also revealed that the age of male medflies had a significant effect on the sexual signalling rate (*F* = 4.902, df = 9, 1179, *P* < 0.001) (Fig 3). Diet alteration from YS to S dramatically reduced the frequency of sexual signalling (less than 4 out of 13 observations). Sexual signalling activity of YS → S fed males followed a gradual decrease until the age of 60 days when the frequency levels reached those of sugar-only (S) control males (Tukey’s HSD test *P* > 0.05). Reversely, diet alteration from S to YS increased directly the frequency of sexual signalling (more than 4 out of 13 observations). The respective increase was immediate and 10 days after the diet alteration it reached the levels of full-diet (YS) control males (Tukey’s HSD test *P* > 0.05) (Fig 3). Until the age of 50 days, sexual signalling rates of males subjected to the YS → S dietary protocol were almost half than that of the full-diet (YS) control males (Tukey’s HSD test *P* < 0.05) and slightly higher than that of sugar-only (S) control males (S2 Fig). On the contrary, males subjected to the S → YS dietary protocol performed sexual signalling as often as full-diet (YS) control males, and more frequent than sugar-only (S) (control) males (Tukey’s HSD test *P* < 0.05). Already 10 days after diet alteration, S → YS males achieved an almost two-fold immediate increase of their signalling than sugar-only (S) control males (S2 Fig).

Taking together lifespan and sexual signalling, our data showed that full-diet fed males (YS) lived longer and performed high levels of sexual signalling whereas sugar-only fed males (S) lived shorter and had a low sexual performance (Figs 2 and 3, Table 1). Both longevity and sexual signalling rates were similar between YS → S and S males. Hence, early-life access to protein is not sufficient to increase male fitness. On the contrary, S → YS males exhibited similar trends of sexual signalling but lower survival rates compared to YS males, especially until the age of 50–80 days of adult life. Within 20 days post PS, S → YS males increased sexual signalling rates to levels similar to YS fed ones (Fig 3). However, mortality rates of S → YS males at the above time window were similar to S rather than to YS males.

## Discussion

Our data revealed that diet quality early in life (first 15 days of adult life) has a crucial effect on two main fitness traits of male medflies: survival and sexual signalling. Protein availability only during the first 15 days of adult life (YS → S) is not sufficient in maintaining high survival and sexual signalling rates at advanced ages. Mortality rates of males subjected to PS early in adult are similar to sugar-only fed males and gradually reduced later with the provision of a proteinaceous diet. Interestingly, in this latter case, dietary improvement also causes an intense and immediate increase of sexual signalling that remains in high frequencies even in extreme ages. Protein availability after the age of 15 days seems to be invested in sexual performance on the expense of longevity for the majority of individuals.

In agreement with our findings, earlier studies revealed higher mortality rates of males fed on a sugar-only diet compared to those fed a full diet (yeast hydrolysate plus sugar) [26, 27, 36]. The proportion of yeast hydrolysate in the adult diet may be a major determinant of male life span. For example, in an older study not mated, laboratory adapted, male medflies, maintained in groups, lived longer when had access to a diet containing 2% and 5% compared to 0 (sugar only) and 25% yeast hydrolysate [7]. Apparently, a list of factors, such as insect strains (laboratory flies are usually short-lived) and crowding holding conditions (grouped caging leads to antagonistic/ aggressive behaviours for access to food, mates, water and space) can severely affect the experimental outcome [37, 38]. Additionally, dietary protocols differ significantly amongst scientific groups in terms of quality (protein availability, sugar, vitamins etc.), quantity and proportion of nutrients [6]. In the present study, dietary manipulation included the total exclusion of yeast hydrolysate from the diet which consists a proteinaceous nutriment, rich in essential amino acids that contribute to lifespan extension [5], and a source of vitamins and minerals. The availability of plain granulated sugar (sucrose) provides exclusively hydrocarbons and none of the nutrients (vitamins, minerals, fatty acids, trace elements) that in combination to protein (amino acids) seem to contribute to lifespan extension. The current study, however, has not addressed (this was out of the scope) possible effects of vitamins or other micronutrients that may be present in yeast hydrolysate on male longevity and sexual performance. A different experimental protocol employing chemically defined or holidic diets is required in future studies to validate the precise role of protein, vitamins and micronutrients on regulating lifespan and sexual signalling in this species [39].

Protein starvation after attaining sexual maturity (YS → S) reduced the lifespan of male medflies to similar levels of sugar-only fed (S) males. It seems that protein availability during the first 15 days of male life was not sufficient in maintaining survival at high levels, despite that the energy demanding behaviour of sexual signalling remained at relatively low levels. Hence, a constant or regular access to a diet of high nutrient value is essential for sustaining longer life span in male medflies.

Male medflies with constant access to a YS diet exhibit sexual signalling in higher frequencies than males fed a S diet [28, 32], participate more often in leks [24], exhibit frequent courtship behaviour [23], and inhibit the sexual receptivity of mated females [40]. In the present study, protein availability before attaining sexual maturity (<15 days) (YS → S), contributed in maintaining slightly higher frequency of sexual signalling until the age of 50 days, compared to the performance of sugar-only (S) control males. Apparently, available nutrients in yeast hydrolysate that males accumulate during the first 15 days of adult life are invested in reproductive activities such as sexual signalling in succeeding ages (until the age of 50 days). Carbohydrate, glycogen and lipid reserves have been positively correlated with the reproductive success of male medflies in the past [41]. It is important to notice in our study that early-life availability of a proteinaceous intake can be stored, preserved and provide a reproductive benefit later in life (until the age of 50 days).

Protein starved males (S → YS) exhibited high frequency (similar to YS fed males) of sexual signalling within 5 days after diet switch. However, their survival rate followed similar patterns to S fed males until approximately day 70. Previous studies have shown that male medflies provided with several dietary protocols (including a sugar-only diet) attain sexual maturity until the age of 10–12 days [28, 32]. Hence, the immediate response of protein starved males to protein availability, expressed as an increase of sexual signalling activity, could not be related to sexual maturation process. In fact, increase in sexual signalling after switching to protein rich diet may indicate that: (a) sexual signalling is an energy consuming behaviour depending on the nutritional level of the individual expressing it [41, 42], (b) nutrient metabolism and consumption is a rather rapid procedure, and (c) males probably allocate nutrients in sexual performance rather than soma maintenance. The latter conclusion is also supported by the low survival rates of S → YS males until the age of 70 days. Although the underlying physiological mechanism involved in this process is not yet known, the reduction in the frequency of sexual signaling in advanced ages may account for the allocation of nutrient and energy to soma maintenance.

A small proportion of males that were subjected to the S → YS protocol reached a more advanced age and exhibited sexual signalling more often especially in older ages compared to full-diet (YS) control males. This finding may indicate that reduced reproductive effort in young ages of PS males is similar to the “waiting mode” of female medflies proposed by Carey and co-workers [25]. Testing the effect of different types of DM on the interaction between sexual signalling and longevity would shed important light on the cost of exhibiting energy consuming behaviours and its relation to male fitness.

In conclusion, protein availability only early in adult life is not sufficient to support lifetime energy-consuming behaviours such as sexual signalling and cannot sustain high survival rates after switching to a sugar only diet. This finding seems to contrast the theory of lifespan expansion through DR, leastwise when diet is applied with the nutritional protocol followed in the present study. On the other hand, access to a proteinaceous diet after attaining sexual maturity, “restores” shortly the sexual signalling performance and progressively reduces the detrimental effects of protein elimination on survival. Exploring the underlying mechanisms that lead to different results when different dietary protocols are applied and assessing the effect of several DM regimes on other biological and behavioural traits (male mating competitiveness, copula duration, sperm production and transfer etc.) would provide interesting information and contribute towards better understanding the physiological and behavioural shifts that DM triggers.

## Supporting information

S1 Dataset File Dataset.xls file with raw data. (XLS)

S1 Fig Fitted Weibull survival (a), hazard rates (b) and density estimate (c) of males subjected to 4 different dietary regimes (YS–orange line, S → YS–red line, S–blue line, YS → S–green line) in relation to age.
(TIF)

S2 Fig Proportion of mean daily sexual signalling of males subjected to YS → S diet (continuous line), of males subjected to S → YS diet (dotted line) and of control males continuously fed with either S or YS (grey line) divided by the mean daily sexual signalling of males continuously fed a YS diet (a) and by the mean daily sexual signalling of males continuously fed a S diet (b).
(TIF)

S1 Table Parameter estimates for male lifespan of Weibull model distribution with lower and upper 95% CI for the 4 dietary regimes. (PDF)
