# Supplementary figures and images for: Effects of early-life protein starvation on longevity and sexual performance of male medfly
Source: PLoS One. 2019 Jul 25;14(7):e0219518. doi: 10.1371/journal.pone.0219518 (PMC6657835; doi:10.1371/journal.pone.0219518)

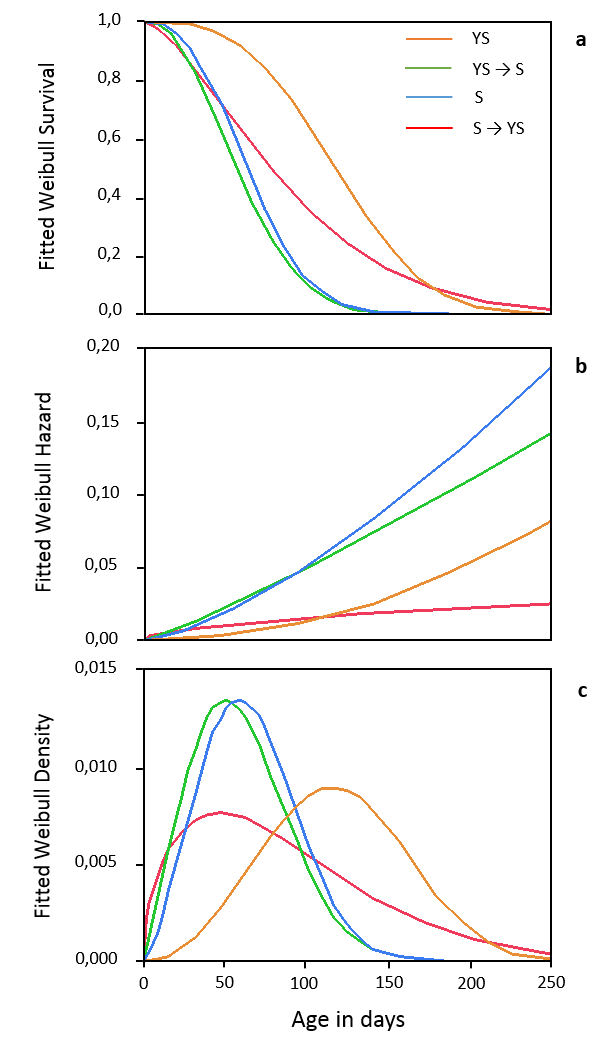

Supplement: S1 Fig — Fitted Weibull survival (a), hazard rates (b) and density estimate (c) of males subjected to 4 different dietary regimes (YS–orange line, S → YS–red line, S–blue line, YS → S–green line) in relation to age. (TIF) [file pone.0219518.s002.tif]

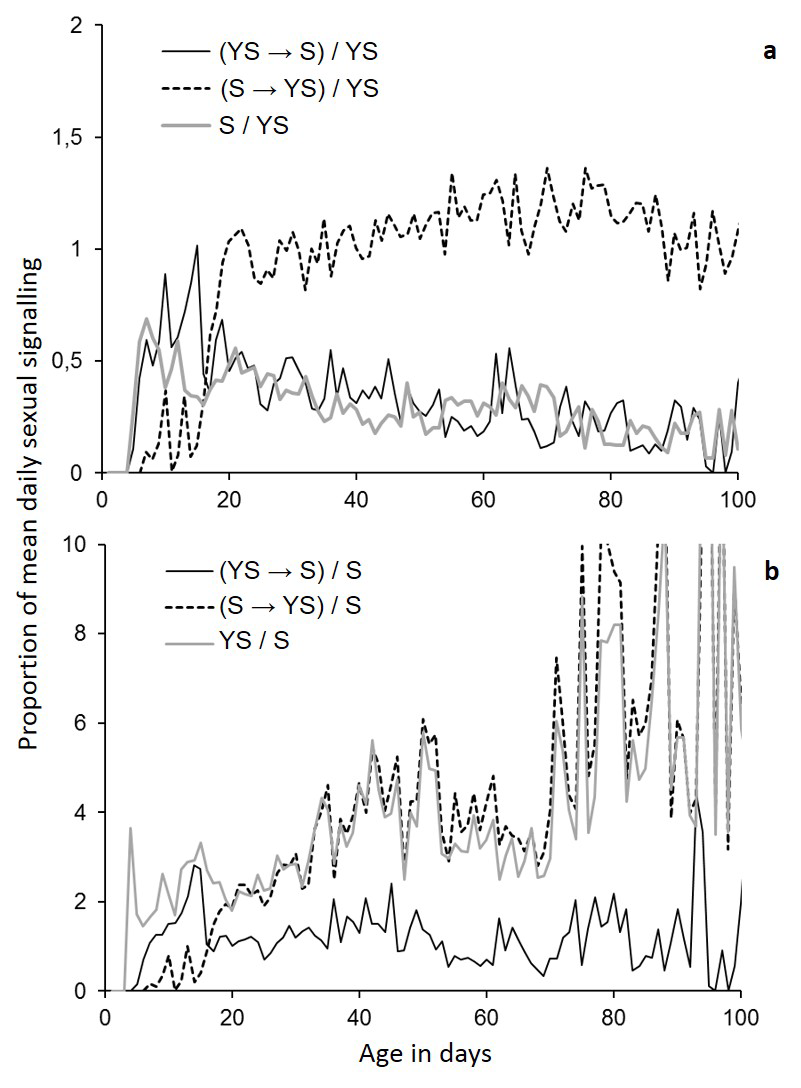

Supplement: S2 Fig — Proportion of mean daily sexual signalling of males subjected to YS → S diet (continuous line), of males subjected to S → YS diet (dotted line) and of control males continuously fed with either S or YS (grey line) divided by the mean daily sexual signalling of males continuously fed a YS diet (a) and by the mean daily sexual signalling of males continuously fed a S diet (b). (TIF) [file pone.0219518.s003.tif]
