# Supplementary material for: Effects of early-life protein starvation on longevity and sexual performance of male medfly
Source: PLoS One. 2019 Jul 25;14(7):e0219518. doi: 10.1371/journal.pone.0219518 (PMC6657835; doi:10.1371/journal.pone.0219518)
Supplement: S1 Table — (PDF) [file pone.0219518.s004.pdf]

Source of variance		Parameter	
Diet	n	scale α	shape β
YS	141	132.36 (124.84 – 140.14)	3.01 (2.62 – 3.43)
YS → S	42	67.45 (57.69 – 78.29)	2.14 (1.66 – 2.69)
S	147	73.48 (68.39 – 78.82)	2.42 (2.12 – 2.74)
S → YS	47	98.26 (79.59 – 120.15)	1.50 (1.17 – 1.87)
